# Investigating the relationship of COVID-19 preventive and mitigation measures with mosque attendance in Pakistan

**DOI:** 10.1371/journal.pone.0294808

**Published:** 2023-12-04

**Authors:** Hamza Umer, Muhammad Salar Khan

**Affiliations:** 1 Hitotsubashi Institute for Advanced Study (HIAS), Hitotsubashi University, Tokyo, Japan; 2 Institute of Economic Research (IER), Hitotsubashi University, Tokyo, Japan; 3 Schar School of Policy and Government, George Mason University, Arlington, Virginia, United States of America; Centro Escolar University, PHILIPPINES

## Abstract

Social distancing served as a principal strategy to curtail the spread of COVID-19. However, congregational activities in mosques made it challenging to practice social distancing and led to a rapid surge in virus infections in several Muslim countries. This study uses nationally representative cross-sectional data from Pakistan, a Muslim-majority country, to examine the relationship of practicing preventive measures (such as social distancing, wearing mask and hand washing) and mitigation measures (like avoid going to the market, social gatherings, healthcare seeking, use of public transport, and long-distance travel) with mosque visits by utilizing logistic regressions. The results show that individuals adhering to preventive and mitigation measures also avoid visiting mosques and other religious gatherings. From a policy perspective, these results suggest that the government of Pakistan can avoid direct religious confrontation when it needs to minimize mosque visits to curtail the spread of the virus by implementing preventive and mitigation measures.

## 1. Introduction

The COVID-19 pandemic recently engulfed the world, causing socio-economic disruptions in many countries. Although there has been a gradual recovery, public gatherings, including those in religious places such as mosques and churches, remain a concern for the spread of the virus. To control its spread, preventive and mitigation strategies such as social distancing, mask-wearing, hand-washing, and avoiding public spaces have been touted as effective mechanisms [1,2]. This paper aims to investigate the role of preventive and mitigation measures on mosque attendance in Pakistan.

Given that congregational prayers and other communal activities in mosques may pose a risk for the spread of COVID-19, the study answers the question whether COVID-19 preventive and mitigation measures relate with mosque attendance in Pakistan. Although the government and various Islamic organizations and scholars have taken steps to control the spread of the virus, but some religious clergy continued to hold large congregational prayers and masses in mosques. Therefore, this study seeks to explore the relationship between individual observance of COVID-19 preventive and mitigation measures and mosque visits. The study is motivated by the need to control the spread of COVID-19 in Pakistan and to find ways to improve citizens’ voluntary non-participation in religious activities without directly confronting religious groups.

Most religions prescribe congregations as part of their practice of faith. Islam also emphasizes and commands congregational prayers. For instance, Muslims are commanded to offer five mandatory daily and Friday prayers in mosques while standing in rows close to each other. In normal times, congregational prayers and other communal activities pose no serious threat to the health of attendees. However, during the pandemic, these congregations, like any other gatherings, can serve as breeding grounds for the spread of COVID-19 and pose serious medical challenges to attendees and other people in their proximity. The evidence supports this preposition, with examples including a rapid surge in COVID-19 cases in Iran linked to uncontrolled shrine attendance [3], a six-day religious gathering in Malaysia leading to spread [4,5], and similar masses in Pakistan and India contributing to rapid virus transmission [1]. Attendance in churches has also been correlated with virus spread during the pandemic [6]. For a comprehensive analysis of how religious gatherings have led to virus spread, please see the review article by Lee et al. [7].

Pakistan is predominantly a Muslim country with a population of 96.3% Muslim. Mosques in Pakistan play a pivotal role in observance of congregational prayers, religious events, and, often, communal activities. However, the proximity of activities in mosques can offer potential grounds for the dispersal of COVID-19. The government of Pakistan initially imposed a ban on communal gatherings and restricted the number of people in congregational prayers in mosques to combat the virus spread. At the beginning of the pandemic, the Pakistan Ulema Council (PUC), comprising notable *ulemas* (religious scholars), also issued a *fatwa* (Islamic injunction) to postpone religious and political gatherings, instructing the citizens to observe the COVID-19 guidelines issued by the government and follow COVID-19 preventive measures while offering the congregational Friday prayers [8].

In line with restrictions and guidelines about religious rituals during COVID-19 in other countries [9–11], several other prominent Pakistani Muslim scholars (*Mawlānās*), including Taqi Usmani, Tariq Jameel, and Tahirul Qadri, also urged people to follow the government’s guidelines while offering prayers in mosques [12,13]. Furthermore, the president of Pakistan requested the Supreme Ulema Council (SUC) at Al-Azhar university in Egypt to issue an Islamic verdict (fatwa) related to the COVID-19 guidelines. The council gave a clear ruling to avoid congregational prayers in mosques as a precautionary measure to control the virus spread [14,15]. At a provincial level, the Sindh government banned all congregational prayers in mosques, including the Friday prayer. All the aforementioned steps by the government and religious rulings by Islamic organizations and scholars might have collectively played an essential role in shaping the voluntary non-attendance of congregational prayers and mosque visits.

Despite various steps taken by the government and decrees issued by religious scholars, a significant fraction of the religious clergy continued to hold large congregational Friday prayers in mosques (e.g., Abdul Aziz at the Red Mosque in Islamabad) [16]. Moreover, support for congregational prayers grew further near the holy month of fasting (*Ramadan*). A group of notable religious scholars, including Muneeb-ur-Rehman and Shah Owais Noorani, also joined the call for the resumption of congregational prayers during Ramadan and constituted a 12-member committee to negotiate with the government to exempt mosques from lockdown policies [10,17,18]. This response of religious scholars might have triggered active participation in religious gatherings and congregational prayers in mosques.

Although COVID-19 is largely controlled now, Pakistan’s fragile medical and economic systems are still precarious. Any infectious virus, if allowed to spread through large religious congregations or other means, will cause havoc in the country. In the absence of strict government intervention, the divided stance of religious scholars about religious attendance in the instance of a fatal pandemic, and uneven observance of preventive measures at mosques, voluntary non-participation of citizens in congregational (religious) activities could serve as an effective mechanism to control the massive spread of COVID-19 (or future pandemics), among other mechanisms. The critical problem is how the government can improve citizens’ voluntary non-participation without directly confronting religious groups and clergy. One possible solution is to examine whether individual observance of COVID-19 preventive and mitigation measures relates to mosque visits and participation in other religious activities.

While the existing literature examines several other factors that can play a significant role in controlling the spread of the virus, it lacks an analysis of the association (or relationship) between preventive and mitigation measures and mosque attendance. For example, Dahlan et al. [19] examine the application of the Islamic principle of *hifz al-nafs* in Indonesia to prevent the spread of COVID-19. Al-Astewani [20] examines the role of religious authority and mosque closure in England. Similarly, several studies investigate the effect of social preferences on the observance of COVID-19 preventive behaviors (for example, see studies [21–28]). Our paper adds to this body of literature by analyzing the association of preventive (and mitigation) measures with mosque attendance during the COVID-19 pandemic in Pakistan.

If people who adhere to COVID-19 preventive and mitigation measures also avoid visiting mosques and religious gatherings, the outcome can offer an important avenue through which government can indirectly yet effectively nudge the behavior of mosque attendees through the effective implementation of preventive and mitigation measures. Such an intervention will lead to a win-win situation for both the government and the general public and help mitigate the spread of COVID-19. Hence the question under investigation in this paper is of utmost social and practical importance.

Specifically, the article uses nationally representative data from Pakistan (n = 22,616) to examine whether people who observe preventive and mitigation measures have higher odds of avoiding attendance at mosques and other religious activities. The results suggest that encouraging these measures can indirectly push people to avoid going to mosques and other gatherings, helping to mitigate the spread of the virus without imposing direct restrictions on religious practices. This indirect interjection can be extremely useful in countries like Pakistan, where imposing direct restrictions on the practice of religion can lead to social unrest and backfire.

## 2. Methodology and data details

The analysis in this study relies on the secondary cross-sectional data collected by the Pakistan Bureau of Statistics (PBS). PBS administered an electronic tablet-based survey from October 20, 2020, till November 5, 2020, to examine the impact of the COVID-19 on socio-economic wellbeing during the acute stage of the COVID-19 (April to July 2020) in Pakistan. The survey was administered to a nationally and provincially representative sample of 6,000 households. The sample size was determined based on information about average household consumption as well as food insecurity collected in the Household Integrated Economic Survey 2018–19. A two-stage stratified random sampling mechanism was used to select the households. In the first stage, primary sampling units (PSUs) were selected using the random sampling proportional size method. This resulted in the selection of 500 PSUs of which 349 were urban (about 70%) and 151 were rural (about 30%) areas. In the second stage, 12 households were randomly selected from each PSU, leading to the final sample of 6,000 households (about 70% urban and 30% rural). The 70–30 ratio of urban and rural respondents was chosen primarily because the spread of COVID-19 and its effects were higher in the urban areas.

The survey questions were designed in consultation with stakeholders, including the Food and Agricultural Organization (FAO), the World Bank, the United Nations Development Program (UNDP), the World Health Organization (WHO), and the Ministry of Planning, Development and Special Initiatives (MOPDSI). The survey had eight major sections that elicited demographic information, the impact of COVID-19 on employment and food security, participation in social assistance programs, housing characteristics, financial coping strategies, household assets, practices to mitigate and prevent the spread of the COVID-19, and the reduction in the usage of health services due to the pandemic.

We should note that IRB (Institutional Review Board) approval and participation consent are inapplicable since we use data collected by PBS. The data and survey used in the study are publicly available on the PBS website at: https://www.pbs.gov.pk/content/microdata-covid-19. The STATA do file used for the analysis will be readily provided upon request.

To perform the analysis, we merged data from three relevant modules of the survey: Module A (demographic information), Module B (containing income and employment information) and Module I (containing information about mosque attendance and the preventive and mitigation measures). The final dataset by merging these three relevant sections of the COVID-19 survey consists of usable 22,616 observations from 5,507 households (average number of observations per household = 4.11). The dependent variable is a binary outcome that examines whether respondents (10 years or older) avoid going to mosques or religious gatherings because of COVID-19. The primary independent variables consist of a set of three preventive measures (social distancing, wearing mask, and washing hands) and a set of five mitigation measures (avoid going to the market, social gatherings, healthcare seeking, use of public transport, and long-distance travel) observed by respondents (10 years or older) to safeguard themselves from the virus. The separate classification of preventive and mitigation measures follows from the survey. Moreover, the response for preventive measures is based on a modified Likert scale with four options, while the response for mitigation measures is binary. These disparate response scales also naturally suggest separately classifying (and analyzing) preventive and mitigation measures.

We also use several adjustment variables that capture the basic demographic variations (age, gender, marital status, education), income differences as well as regional differences (urban versus rural). We also include provincial dummies to control for the provincial fixed effects. A quick look at the control variables shows that the data have a well-balanced representation of both male (almost 52%) and female subjects, with an average age of almost 31 years. Most respondents (nearly 54%) are married, and the sample is diverse in educational backgrounds and has respondents with no formal education, as well as those with PhD degrees. Almost 30% of respondents are from rural areas. No income information is available for a significant proportion of respondents (almost 65%), either because they do not fall into the working age or because people do not report their income. Thus, income is added as an *extended adjustment variable* in analysis to preserve the number of observations. Further data details are in Table 1.

**Table 1:** Data summary.

Variable	Observations
**Dependent Variable**	
Avoid going to Mosque / Religious Gatherings	22,616
Yes (1)	17,503
No (0)	5,113
**Preventive Measures**	
***1) Social Distancing***	
Always while outside	11,452 (50.63%)
Sometimes when outside	5577 (24.66%)
Rarely when outside	3144 (13.90%)
Never	2444 (10.81%)
***2) Wearing Mask***	
Always while outside	11,633 (51.43%)
Sometimes when outside	5586 (24.70%)
Rarely when outside	2880 (12.73%)
Never	2518 (11.13%)
***3) Hand Washing (during 24 hours)***	
Always while outside	11,948 (52.83%)
Sometimes when outside	5877 (25.99%)
Rarely when outside	3192 (14.11%)
Never	1599 (7.07%)
**Mitigation Measures**	
***1) Avoid going to the market***	
Yes	18,635 (82.40%)
No	3981 (17.60%)
***2) Avoid social gathering of more than 4 people***	
Yes	17,957 (79.40%)
No	4659 (20.60%)
***3) Avoid healthcare seeking***	
Yes	18,893 (83.54%)
No	3723 (16.46%)
***4) Avoid public transport***	
Yes	19,236 (85.05%)
No	3380 (14.95%)
***5) Avoid long distance travel***	
Yes	19,557 (86.47%)
No	3059 (13.53%)
**Main Adjustment Variables**	
Male	11,750 (51.95%)
Age	22,617 (Mean = 31.17 Years; SD = 16.36)
*Marital Status*	
Never married	9,385 (41.50%)
Currently married	12,303 (54.40%)
Widow / Widower	757 (3.35%)
Divorced	72 (0.32%)
Separated	36 (0.16%)
Married but girl still lives with parents	64 (0.28%)
*Maximum Education*	
No formal education	7813 (34.54%)
Nursery	169 (0.75%)
Kindergarten	1465 (6.48%)
Primary	3581 (15.83%)
Middle	2688 (11.88%)
Matric	3162 (13.98%)
Intermediate	1908 (8.44%)
Degree in Engineering	91 (0.40%)
Degree in Medicine	65 (0.29%)
Degree in Computer	33 (0.15%)
Degree in Agriculture	5 (0.02%)
Degree in other subjects	966 (4.27%)
Master	603 (2.67%)
M.Phils.	41 (0.18%)
PhD	27 (0.12%)
*Region*	
Rural	6858 (30.32%)
Urban	15,759 (69.68%)
*Province*	
KPK	3396 (15.02%)
Punjab	7708 (34.08%)
Sindh	6348 (28.07%)
Baluchistan	2983 (13.19%)
AJ&K	1189 (5.26%)
Gilgit-Baltistan	993 (4.39%)
**Extended Adjustment Variable**	
ln (Monthly Income)	7829 (Mean = 8.84; SD = 3.00)

## 3. Empirical analysis

The following two specifications are used to examine the relationship of preventive and mitigation measures with avoiding visiting mosques and other religious congregations.


$$Y_{i}=\beta_{0}+\beta_{1}\;Social\;Distancing+\beta_{2}\;Wearing\;Mask+\beta_{3}\;Hand\;Washing+\beta_{4}\;X_{i}+\epsilon_{i}$$ (1)

$$Y_{i}=\beta_{0}+\beta_{1}\;Avoid\;Market+\beta_{2}\;Avoid\;Social\;Gathering+\beta_{3}\;Avoid\;Healthcare+\beta_{4}\;Avoid\;Public\;Transport+\beta_{5}\;Avoid\;Long\;Travel+\beta_{6}\;X_{i}+\epsilon_{i}$$ (2)

Whereas *Y*_*i*_ is the dependent variable (avoid visiting mosque) for individual *I*, the main independent variables of interest are three preventive measures in Eq (1) and five mitigation measures in Eq (2). The preventive and mitigation measures are examined in separate specifications because they examine different dimensions of precautions and use different scales to elicit responses. A positive and significant coefficient for the main explanatory variables would mean respondents who observe preventive or mitigation measures also avoid visiting mosques or religious gatherings. The vector *X*_*i*_ in both equations is a set of adjustment variables (both main and extended adjustment variables), while *ϵ*_*i*_ represents the random error term. As the dependent variable is binary, Eqs 1 and 2 are estimated using logistic regressions, and the analysis is performed using STATA 16.

## 4. Results

The statistical results with the dependent variable (avoid going to mosque/religious gatherings) and the set of preventive measures are reported in Table 2. In contrast, these results with the set of mitigation measures are reported in Table 3. For the sake of brevity, results for primary explanatory variables are reported in the main text, while the complete results incorporating the full set of main and extended adjustment variables are reported in S1 and S2 Tables, respectively. Tables 2 and 3 present outputs from three different regression models. The first model includes results from regressions that use only the primary explanatory variable, and the second model includes results from regressions with main adjustment variables. In contrast, the third model includes outcomes from regressions with main and extended adjustment variables (i.e., log of income). These three regressions are conducted primarily to examine the robustness of coefficients.

**Table 2:** Preventive measures & mosque attendance (logistic regressions, odds ratios).

	Avoid Visiting Mosque	Avoid Visiting Mosque	Avoid Visiting Mosque
Model #	[1]	[2]	[3]
**Social Distancing** (Base: Never)			
Always	3.376***	2.220***	2.461***
	(2.773–4.109)	(1.802–2.734)	(1.682–3.600)
Sometimes	2.200***	1.451***	1.762***
	(1.862–2.599)	(1.215–1.732)	(1.249–2.485)
Rarely	1.570***	1.270***	1.639***
	(1.336–1.845)	(1.072–1.504)	(1.162–2.313)
**Wearing Mask** (Base: Never)			
Always	0.887	1.057	1.132
	(0.712–1.106)	(0.838–1.335)	(0.756–1.696)
Sometimes	0.662***	0.840*	1.091
	(0.552–0.795)	(0.692–1.019)	(0.761–1.563)
Rarely	0.655***	0.679***	0.899
	(0.555–0.773)	(0.569–0.810)	(0.627–1.288)
**Hand Washing** (Base: Never)			
Always	8.555***	10.574***	14.935***
	(7.013–10.437)	(8.559–13.063)	(9.958–22.399)
Sometimes	5.484***	6.551***	7.508***
	(4.606–6.528)	(5.439–7.890)	(5.172–10.900)
Rarely	3.402***	3.850***	4.019***
	(2.845–4.069)	(3.186–4.652)	(2.736–5.904)
Observations	22,616	22,611	7,827
Main Adjustment Variables	No	Yes	Yes
Extended Adjustment Variable	No	No	Yes
Pseudo R-Squared	0.153	0.220	0.251

95% Confidence intervals in parentheses.

*** p<0.01

** p < 0.05

* p <0.10.

**Table 3:** Mitigation measures & mosque attendance (logistic regressions, odds ratios).

	Avoid Visiting Mosque	Avoid Visiting Mosque	Avoid Visiting Mosque
**Model #**	**[1]**	**[2]**	**[3]**
**Avoid Going to Market**	4.611***	5.045***	4.918***
	(4.118–5.164)	(4.472–5.692)	(4.137–5.846)
**Avoid Social Gatherings**	5.745***	4.941***	3.959***
	(5.135–6.427)	(4.381–5.572)	(3.290–4.762)
**Avoid Healthcare Seeking**	3.020***	3.137***	2.901***
	(2.624–3.477)	(2.708–3.634)	(2.348–3.584)
**Avoid Public Transport**	1.098	0.932	0.844
	(0.915–1.319)	(0.770–1.127)	(0.645–1.104)
**Avoid Long Distance Travel**	1.459***	1.995***	1.265*
	(1.216–1.750)	(1.652–2.411)	(0.962–1.661)
Observations	22,616	22,611	7,827
Adjustment Variables	No	Yes	Yes
Extended Adjustment Variable	No	No	Yes
Pseudo R-Squared	0.385	0.440	0.385

95% Confidence intervals in parentheses.

*** p<0.01

** p < 0.05

* p <0.10.

The results for preventive measures in Table 2 indicate that, *ceteris paribus*, respondents who always, sometimes, or rarely practice social distancing or washing hands have higher odds of avoiding visiting mosques or religious gatherings (ranging from 1.3 times to 15 times higher) compared to those who never do. These results remain significant in all three regression models. However, in comparison to respondents who never wear a face mask, those who either sometimes or rarely wear a mask (Models 1 and 2) have lower odds of avoiding visiting mosques, *ceteris paribus*. Nevertheless, these results are not stable and become insignificant once income is adjusted for (Model 3).

The results for mitigation measures in Table 3 show that individuals who avoid going to the market, social gatherings, seeking healthcare, and long-distance travel have higher odds (ranging from 1.3 to 5.7 times) of avoiding visiting mosques or other religious gatherings, ceteris paribus. The results remain significant (and positive) irrespective of the regression specification. However, for one mitigation measure (avoid public transport), the coefficient is insignificant in all three regressions. By and large, the results are unidirectional and provide sufficient evidence that individuals observing mitigation measures against COVID-19 have lower odds of visiting mosques for congregational activities.

Apart from the main explanatory variables, several adjustment variables also have significant coefficients in regressions with preventive and mitigation measures. For the sake of brevity, only adjustment variables with significant coefficients in both Eqs 1 and 2 are mentioned here, while complete results are available in S1 and S2 Tables. It is found that male respondents have lower odds (about half) of avoiding going to the mosque than females, ceteris paribus. There is also some evidence that respondents with basic education (i.e., kindergarten), or higher education (i.e., MSc) have higher odds of avoiding going to mosques than respondents with no formal education, ceteris paribus. Although this result does not demonstrate a linear relationship between education and avoiding mosque visits, it suggests that educated people to some extent have higher odds of abstaining from religious activities that pose a risk of COVID-19 infection. Additionally, there is evidence that respondents in rural areas have lower odds of avoiding visiting mosques compared to those in urban areas, ceteris paribus. Finally, compared to the Punjab province, respondents in all other provinces have lower odds of avoiding mosque visits.

## 5. Robustness check

Since the preventive measures examine different aspects of the same reality, there is a possibility that these measures are correlated with each other. Similarly, the mitigation measures examine various aspects of behavior towards COVID-19 prevention and can be associated with each other. A pairwise correlation analysis reveals significant correlations (p<0.01) among three preventive measures and five mitigation measures (output reported in S3 Table). Therefore, to avoid multicollinearity in estimated coefficients, we follow the pandemic literature [29] and perform a principal component analysis (PCA) to construct a prevention index based on a linear combination of three preventive measures and a mitigation index based on a linear combination of five mitigation measures. The analyses with prevention and mitigation indexes are reported in Table 4, while complete results are in S4 Table. The results also indicate that people who observe preventive and mitigation measures have higher odds of avoiding going to mosques, ceteris paribus. In particular, individuals who practice preventive measures are 2.4 to 2.6 times relatively more likely to avoid visiting mosques. Similarly, those who practice mitigation measures are approximately 2.8 to 3.4 times more likely to avoid visiting mosques. The results remain significant and unidirectional, irrespective of whether adjustment variables are excluded or included in regressions, and support the earlier findings.

**Table 4:** Mitigation & preventive measures indexes from PCA and mosque attendance (logistic regressions, odds ratio).

	Avoid Visiting Mosque	Avoid Visiting Mosque	Avoid Visiting Mosque	Avoid Visiting Mosque	Avoid Visiting Mosque	Avoid Visiting Mosque
Model #	[1]	[2]	[3]	[4]	[5]	[6]
						
**Preventive Measures Index**	2.469***	2.447***	2.645***			
	(2.391–2.551)	(2.354–2.544)	(2.470–2.832)			
**Mitigation Measures Index**				3.399***	3.394***	2.796***
				(3.290–3.511)	(3.273–3.518)	(2.647–2.953)
Observations	22,616	22,611	7,827	22,616	22,611	7,827
Main Adjustment Variables	No	Yes	Yes	No	Yes	Yes
Extended Adjustment Variable	No	No	Yes	No	No	Yes
Pseudo R-Squared	0.140	0.212	0.244	0.276	0.349	0.320

95% Confidence intervals in parentheses.

*** p<0.01

** p < 0.05

* p <0.10. Varimax rotation is performed during PCA for both indices.

## 6. Discussion

The study examined the relationship between adherence to preventive and mitigation measures and mosque visits using nationally representative data from Pakistan. The results, including robustness checks, revealed that people who practice social distancing and frequently wash their hands have higher odds of avoiding visiting mosques and other religious gatherings. Similarly, people who avoid going to markets, social gatherings, seeking healthcare, and long-distance travel also exhibit higher odds of avoiding visiting mosques and other religious congregations. These results suggest that people who follow prevention and mitigation measures have higher odds of avoiding religious gatherings and hence contributing towards controlling the spread of the virus.

The findings make sense because mosques and other religious congregations often involve large gatherings of people, which can increase the risk of COVID-19 transmission. Social distancing, frequent hand washing, and avoiding crowded places are some of the key preventive measures recommended by health authorities to limit the spread of the virus. Therefore, people who are more cautious and follow these measures are perhaps more concerned about their health and safety, and consequently, less inclined to attend religious gatherings. Additionally, people who avoid going to markets, social gatherings, seeking healthcare, and long-distance travel may also be more aware of the risks associated with COVID-19 and therefore more tilted to avoid religious gatherings to minimize their risk of exposure to the virus.

The adjustment variables with significant coefficients also merit our attention. Contrary to the results mentioned above, the study found that people who wear masks have lower odds of avoiding mosque visits. One possible reason for this behavior is mask-wearing people may perceive masks to offer complete protection against COVID-19. As for the result that male respondents having lower odds of avoiding mosque visits than females, most possibly indicates that female members of society perceive a higher risk of COVID-19 infection. Even pre-pandemic, the attendance of women in mosques is generally lower than men in Pakistan, and this gap continues to exist during the pandemic.

With respect to regional adjustment variables, the results that urban residents have higher odds of avoiding mosque visits compared to rural residents, most possibly due to the less severe prevalence of COVID-19 and a relative lack of awareness of preventive and mitigation measures in rural areas. Finally, the differential result in the Punjab province compared to other provinces requires a more nuanced analysis, but stricter enforcement of lockdowns in Punjab could be one reason for fewer mosque visits there.

The existing literature has predominantly examined the link between socioeconomic preferences and COVID-19 preventive strategies. For example, Bargain and Aminjonov [21] found that pre-pandemic trust predicted social mobility in Europe, Campos-Mercade et al. [23] reported a positive link between prosocial behavior and preventive behavior in Sweden, Barrios et al. [22] found that social capital influenced social distancing in the US and Europe, and Umer [27] reported that both trust and prosocial behavior measured just before or even long time before the pandemic predicted preventive behavior in the Netherlands. While the direction of results from these somewhat similar studies parallels our study’s findings, most of these studies have focused on developed countries with predominantly Christian populations. Our findings contribute to this stream of literature and add another dimension by exploring how adherence to preventive and mitigation measures relates with mosque attendance and consequently helps to mitigate COVID-19. Our findings bring forth a much-needed perspective from a developing Muslim country and offer a unique solution to mitigate the pandemic by focusing on a different aspect of human behavior.

The current findings, along with existing studies, highlight that there can be multiple ways to nudge human behavior to mitigate infectious diseases such as COVID-19. However, the tools used in developed and non-Muslim countries to mitigate COVID-19 might not be directly applicable to developing, Muslim majority countries like Pakistan where congregational worship in mosque occupies a pivotal position. Therefore, we need to rely on a shrewd approach that does not lead to a direct confrontation with religious sentiments of the public, and at the same time, is effective in promoting preventive behaviors, including limited mosque visits during highly infectious disease outbreaks.

## 7. Policy implications

The study’s results have significant policy implications for countries like Pakistan where religion and social gatherings are deeply ingrained in culture and society. Therefore, strict restrictions on religious activities can lead to social unrest. Pandemics, however, necessitate the government to impose strict measures such as lockdowns and a ban on religious gatherings to control the spread of the disease. Our results show that the government can indirectly control participation in religious gatherings by using subtle nudges to encourage people to adhere to preventive and mitigation measures. This approach can be more effective in mitigating the spread of the virus without causing religious unrest.

Moreover, the study’s implications extend beyond religious gatherings to other social activities and gatherings. Adherence to preventive and mitigation measures can potentially reduce participation in all types of social activities and help control the spread of the disease. Thus, policymakers should consider the spillover effects of encouraging preventive and mitigation measures on reducing both religious and social participation.

Lastly, the implications of this study can be relevant to other culturally similar countries such as India or Muslim-dominant countries, including Bangladesh, Indonesia, and Malaysia. These countries also struggled to contain the virus spreading through religious (and social) gatherings. Policymakers in these countries can learn from the findings of this study and adopt similar policies to control the spread of the virus.

## 8. Conclusion

The study analyzed nationally representative data from Pakistan to investigate the relationship between preventive and mitigation measures and mosque visits. The results indicate that individuals who practice social distancing, hand washing, and avoid public spaces, as well as adhere to other measures, have higher odds of avoiding mosque visits and other religious gatherings, which can aid in controlling the spread of the virus. Policymakers need to be aware that interventions to contain the spread of COVID-19 at worship places do not necessarily have to rely on direct, stringent, and publicly unpopular restrictions on religious practice.

We discuss some limitations of the study. First, social desirability bias can potentially influence self-reported participation in religious activities, mosque attendance, as well as self-reported observance of the mitigation and prevention measures, thereby causing a bias in the outcomes. Second, COVID-19 itself can influence the religiosity of Muslims and subsequently affect their participation in religious activities [30]. Therefore, the influence of adherence to preventive and mitigation measures on mosque attendance and involvement in religious activities can be confounded by the direct or indirect effects of COVID-19 on religiosity.

The study raises other important and interrelated questions for future research, such as whether calling for avoidance of religious gatherings by religious leaders or state institutions is more effective in promoting voluntary non-attendance of mosques, and how direct or indirect contact with COVID-19 affects the religiosity of Muslims and their participation in religious gatherings. Further research in these areas would provide interesting insights and help shed light on effective strategies for controlling the spread of the virus while respecting religious practices and beliefs.

## Supporting information

S1 Table Preventive measures & mosque attendance logistic regressions (odds ratios)–complete results. (DOCX)

S2 Table Mitigation measures & mosque attendance logistic regressions (odds ratios)–complete results. (DOCX)

S3 Table Correlation analysis. (DOCX)

S4 Table Mitigation & preventive measures indexes from pca and mosque attendance logistic regressions (odds ratios)–complete results. (DOCX)
