# Supplementary material for: Investigating the relationship of COVID-19 preventive and mitigation measures with mosque attendance in Pakistan
Source: PLoS One. 2023 Dec 4;18(12):e0294808. doi: 10.1371/journal.pone.0294808 (PMC10695374; doi:10.1371/journal.pone.0294808)
Supplement: S1 Table — (DOCX) [file pone.0294808.s001.docx]

**S1 Table: Preventive Measures & Mosque Attendance**

**Logistic Regressions (Odds Ratios) – Complete Results**

|  | **Avoid Visiting Mosque** | **Avoid Visiting Mosque** | **Avoid Visiting Mosque** |
| --- | --- | --- | --- |
| **Model #** | **[1]** | **[2]** | **[3]** |
|  |  |  |  |
| **Social Distancing** (Base: Never) | | |  |
| Always | 3.376*** | 2.220*** | 2.461*** |
|  | (2.773 - 4.109) | (1.802 - 2.734) | (1.682 - 3.600) |
| Sometimes | 2.200*** | 1.451*** | 1.762*** |
|  | (1.862 - 2.599) | (1.215 - 1.732) | (1.249 - 2.485) |
| Rarely | 1.570*** | 1.270*** | 1.639*** |
|  | (1.336 - 1.845) | (1.072 - 1.504) | (1.162 - 2.313) |
| **Wearing Mask** (Base: Never) | | |  |
| Always | 0.887 | 1.057 | 1.132 |
|  | (0.712 - 1.106) | (0.838 - 1.335) | (0.756 - 1.696) |
| Sometimes | 0.662*** | 0.840* | 1.091 |
|  | (0.552 - 0.795) | (0.692 - 1.019) | (0.761 - 1.563) |
| Rarely | 0.655*** | 0.679*** | 0.899 |
|  | (0.555 - 0.773) | (0.569 - 0.810) | (0.627 - 1.288) |
| **Hand Washing** (Base: Never) | | |  |
| Always | 8.555*** | 10.574*** | 14.935*** |
|  | (7.013 - 10.437) | (8.559 - 13.063) | (9.958 - 22.399) |
| Sometimes | 5.484*** | 6.551*** | 7.508*** |
|  | (4.606 - 6.528) | (5.439 - 7.890) | (5.172 - 10.900) |
| Rarely | 3.402*** | 3.850*** | 4.019*** |
|  | (2.845 - 4.069) | (3.186 - 4.652) | (2.736 - 5.904) |
| Male |  | 0.396*** | 0.433*** |
|  |  | (0.365 - 0.429) | (0.346 - 0.542) |
| Age |  | 1.000 | 0.999 |
|  |  | (0.997 - 1.003) | (0.993 - 1.004) |
| Marital Status (Base: Currently Married) | | | |
| Never Married |  | 1.423*** | 1.172* |
|  |  | (1.278 - 1.585) | (0.983 - 1.396) |
| Widow / Widower |  | 0.908 | 0.609** |
|  |  | (0.730 - 1.129) | (0.394 - 0.941) |
| Divorced |  | 0.828 | 0.594 |
|  |  | (0.432 - 1.590) | (0.239 - 1.476) |
| Separated |  | 1.037 | 0.928 |
|  |  | (0.437 - 2.461) | (0.248 - 3.472) |
| Married but lives with parents |  | 0.985 | 0.973 |
|  |  | (0.558 - 1.739) | (0.449 - 2.108) |
| Education (Base: No Education) | | |  |
| Nursery |  | 0.588*** | 0.741 |
|  |  | (0.400 - 0.866) | (0.385 - 1.427) |
| Kindergarten |  | 1.252*** | 1.232 |
|  |  | (1.063 - 1.474) | (0.881 - 1.725) |
| Primary |  | 1.023 | 0.893 |
|  |  | (0.911 - 1.148) | (0.738 - 1.081) |
| Middle |  | 1.088 | 1.096 |
|  |  | (0.954 - 1.241) | (0.890 - 1.349) |
| Matric |  | 1.082 | 0.969 |
|  |  | (0.955 - 1.225) | (0.807 - 1.164) |
| Intermediate |  | 1.173** | 1.121 |
|  |  | (1.004 - 1.371) | (0.885 - 1.419) |
| Engineering |  | 1.311 | 1.508 |
|  |  | (0.686 - 2.505) | (0.568 - 4.005) |
| Medicine |  | 1.391 | 1.814 |
|  |  | (0.543 - 3.560) | (0.529 - 6.214) |
| Computer Science |  | 2.748 | 1.968 |
|  |  | (0.534 - 14.152) | (0.284 - 13.648) |
| Agriculture |  | - | - |
|  |  |  |  |
| Other Subjects |  | 1.035 | 1.094 |
|  |  | (0.846 - 1.267) | (0.822 - 1.455) |
| MSc |  | 1.435*** | 1.305 |
|  |  | (1.093 - 1.885) | (0.933 - 1.826) |
| M.Phils. |  | 2.013 | 1.942 |
|  |  | (0.582 - 6.956) | (0.423 - 8.925) |
| PhD |  | 0.940 | 1.345 |
|  |  | (0.332 - 2.664) | (0.335 - 5.399) |
| Rural (Base: Urban) |  | 0.844*** | 0.913 |
|  |  | (0.779 - 0.913) | (0.800 - 1.041) |
| Province (Base: Punjab) | |  |  |
| KPK |  | 0.183*** | 0.197*** |
|  |  | (0.162 - 0.205) | (0.162 - 0.238) |
| Sindh |  | 0.429*** | 0.537*** |
|  |  | (0.385 - 0.479) | (0.453 - 0.635) |
| Baluchistan |  | 0.306*** | 0.117*** |
|  |  | (0.269 - 0.348) | (0.096 - 0.142) |
| Gilgit-Baltistan |  | 0.214*** | 0.196*** |
|  |  | (0.180 - 0.255) | (0.149 - 0.258) |
| AJ&K |  | 0.375*** | 0.690* |
|  |  | (0.309 - 0.454) | (0.473 - 1.006) |
| ln (Monthly Income) |  |  | 1.002 |
|  |  |  | (0.980 - 1.024) |
| Constant | 0.382*** | 1.379*** | 0.785 |
|  | (0.342 - 0.427) | (1.117 - 1.702) | (0.520 - 1.185) |
| Observations | 22,616 | 22,611 | 7,827 |
| Adjustment Variables | No | Yes | Yes |
| Extended Adjustment Variable | No | No | Yes |
| Pseudo R-Squared | 0.153 | 0.22 | 0.251 |

95% Confidence Interval in parentheses. *** p<0.01, ** p < 0.05, * p <0.10.
