# Supplementary material for: Investigating the relationship of COVID-19 preventive and mitigation measures with mosque attendance in Pakistan
Source: PLoS One. 2023 Dec 4;18(12):e0294808. doi: 10.1371/journal.pone.0294808 (PMC10695374; doi:10.1371/journal.pone.0294808)
Supplement: S2 Table — (DOCX) [file pone.0294808.s002.docx]

**S2 Table: Mitigation Measures & Mosque Attendance**

**Logistic Regressions (Odds Ratios) – Complete Results**

|  | **Avoid Visiting Mosque** | **Avoid Visiting Mosque** | **Avoid Visiting Mosque** |
| --- | --- | --- | --- |
| **Model #** | **[1]** | **[2]** | **[3]** |
|  |  |  |  |
| **Avoid Going to Market** | 4.611*** | 5.045*** | 4.918*** |
|  | (4.118 - 5.164) | (4.472 - 5.692) | (4.137 - 5.846) |
| **Avoid Social Gatherings** | 5.745*** | 4.941*** | 3.959*** |
|  | (5.135 - 6.427) | (4.381 - 5.572) | (3.290 - 4.762) |
| **Avoid Healthcare Seeking** | 3.020*** | 3.137*** | 2.901*** |
|  | (2.624 - 3.477) | (2.708 - 3.634) | (2.348 - 3.584) |
| **Avoid Public Transport** | 1.098 | 0.932 | 0.844 |
|  | (0.915 - 1.319) | (0.770 - 1.127) | (0.645 - 1.104) |
| **Avoid Long Distance Travel** | 1.459*** | 1.995*** | 1.265* |
|  | (1.216 - 1.750) | (1.652 - 2.411) | (0.962 - 1.661) |
| Male |  | 0.503*** | 0.540*** |
|  |  | (0.456 - 0.555) | (0.420 - 0.694) |
| Age |  | 0.999 | 0.999 |
|  |  | (0.995 - 1.003) | (0.993 - 1.005) |
| Marital Status (Base: Currently Married) | | | |
| Never Married |  | 1.268*** | 1.109 |
|  |  | (1.111 - 1.449) | (0.910 - 1.351) |
| Widow / Widower |  | 0.956 | 0.753 |
|  |  | (0.734 - 1.244) | (0.458 - 1.241) |
| Divorced |  | 1.126 | 0.995 |
|  |  | (0.457 - 2.776) | (0.317 - 3.125) |
| Separated |  | 0.576 | 0.796 |
|  |  | (0.203 - 1.634) | (0.166 - 3.814) |
| Married but lives with parents |  | 0.902 | 0.902 |
|  |  | (0.426 - 1.912) | (0.329 - 2.475) |
| Education (Base: No Education) | | |  |
| Nursery |  | 0.751 | 0.780 |
|  |  | (0.446 - 1.263) | (0.352 - 1.728) |
| Kindergarten |  | 1.517*** | 1.883*** |
|  |  | (1.234 - 1.866) | (1.269 - 2.793) |
| Primary |  | 1.000 | 0.906 |
|  |  | (0.867 - 1.153) | (0.730 - 1.125) |
| Middle |  | 1.211** | 1.240* |
|  |  | (1.031 - 1.421) | (0.980 - 1.568) |
| Matric |  | 1.107 | 1.066 |
|  |  | (0.955 - 1.283) | (0.870 - 1.305) |
| Intermediate |  | 1.208** | 1.364** |
|  |  | (1.004 - 1.453) | (1.051 - 1.770) |
| Engineering |  | 1.615 | 2.268 |
|  |  | (0.743 - 3.510) | (0.701 - 7.336) |
| Medicine |  | 1.690 | 2.226 |
|  |  | (0.625 - 4.571) | (0.611 - 8.113) |
| Computer Science |  | 7.184 | 6.425 |
|  |  | (0.506 - 101.905) | (0.302 - 136.771) |
| Agriculture |  | - | - |
|  |  |  |  |
| Other Subjects |  | 1.110 | 1.280 |
|  |  | (0.881 - 1.398) | (0.937 - 1.747) |
| MSc |  | 1.802*** | 1.620** |
|  |  | (1.300 - 2.497) | (1.109 - 2.368) |
| M.Phils. |  | 2.943 | 1.993 |
|  |  | (0.722 - 11.993) | (0.392 - 10.128) |
| PhD |  | 0.689 | 0.949 |
|  |  | (0.213 - 2.223) | (0.204 - 4.422) |
| Rural (Base: Urban) |  | 0.777*** | 0.769*** |
|  |  | (0.705 - 0.856) | (0.664 - 0.891) |
| Province (Base: Punjab) | |  |  |
| KPK |  | 0.224*** | 0.246*** |
|  |  | (0.194 - 0.259) | (0.198 - 0.305) |
| Sindh |  | 0.533*** | 0.601*** |
|  |  | (0.466 - 0.611) | (0.497 - 0.727) |
| Baluchistan |  | 0.129*** | 0.069*** |
|  |  | (0.112 - 0.149) | (0.056 - 0.086) |
| Gilgit-Baltistan |  | 0.274*** | 0.291*** |
|  |  | (0.222 - 0.337) | (0.213 - 0.398) |
| AJ&K |  | 0.276*** | 0.571*** |
|  |  | (0.223 - 0.342) | (0.376 - 0.868) |
| ln (Monthly Income) |  |  | 1.025** |
|  |  |  | (1.000 - 1.050) |
| Constant | 0.093*** | 0.301*** | 0.457*** |
|  | (0.083 - 0.105) | (0.237 - 0.383) | (0.300 - 0.696) |
| Observations | 22,616 | 22,611 | 7,827 |
| Adjustment Variables | No | Yes | Yes |
| Extended Adjustment Variable | No | No | Yes |
| Pseudo R-Squared | 0.385 | 0.440 | 0.385 |

95% Confidence interval in parentheses. *** p<0.01, ** p < 0.05, * p <0.10.
