# Supplementary material for: Investigating the relationship of COVID-19 preventive and mitigation measures with mosque attendance in Pakistan
Source: PLoS One. 2023 Dec 4;18(12):e0294808. doi: 10.1371/journal.pone.0294808 (PMC10695374; doi:10.1371/journal.pone.0294808)
Supplement: S3 Table — (DOCX) [file pone.0294808.s003.docx]

**S3 Table: Correlation Analysis**

| **Panel A: Preventive Measures** | | | | |  |
| --- | --- | --- | --- | --- | --- |
|  | Social Distancing | Wearing Mask | Hand Washing | |  |
|  |  |  |  |  |  |
| Social Distancing | 1 |  |  |  |  |
| Wearing Mask | 0.8731* | 1 |  |  |  |
| Hand Washing | 0.7966* | 0.8387* | 1 |  |  |
| **Panel B: Mitigation Measures** | | |  |  |  |
|  | Avoid Going to Market | Avoid Social Gatherings | Avoid Healthcare Seeking | Avoid Public Transport | Avoid Long Distance Travel |
|  |  |  |  |  |  |
| Avoid Going to Market | 1 |  |  |  |  |
| Avoid Social Gatherings | 0.6746* | 1 |  |  |  |
| Avoid Healthcare Seeking | 0.6236* | 0.6778* | 1 |  |  |
| Avoid Public Transport | 0.6340* | 0.6788* | 0.7647* | 1 |  |
| Avoid Long Distance Travel | 0.6024* | 0.6438* | 0.7246* | 0.8325* | 1 |

*p < 0.01.
