# Supplementary material for: Investigating the relationship of COVID-19 preventive and mitigation measures with mosque attendance in Pakistan
Source: PLoS One. 2023 Dec 4;18(12):e0294808. doi: 10.1371/journal.pone.0294808 (PMC10695374; doi:10.1371/journal.pone.0294808)
Supplement: S4 Table — (DOCX) [file pone.0294808.s004.docx]

**S4 Table: Mitigation & Preventive Measures Indexes from PCA and Mosque Attendance**

**Logistic Regressions (Odds Ratios) – Complete Results**

|  | **Avoid Visiting Mosque** | **Avoid Visiting Mosque** | **Avoid Visiting Mosque** | **Avoid Visiting Mosque** | **Avoid Visiting Mosque** | **Avoid Visiting Mosque** |
| --- | --- | --- | --- | --- | --- | --- |
| **Model #** | **[1]** | **[2]** | **[3]** | **[4]** | **[5]** | **[6]** |
|  |  |  |  |  |  |  |
| **Preventive Measures Index** | 2.469*** | 2.447*** | 2.645*** |  |  |  |
|  | (2.391 - 2.551) | (2.354 - 2.544) | (2.470 - 2.832) |  |  |  |
| **Mitigation Measures Index** |  |  |  | 3.399*** | 3.394*** | 2.796*** |
|  |  |  |  | (3.290 - 3.511) | (3.273 - 3.518) | (2.647 - 2.953) |
| Male |  | 0.410*** | 0.465*** |  | 0.552*** | 0.560*** |
|  |  | (0.379 - 0.443) | (0.374 - 0.579) |  | (0.506 - 0.603) | (0.444 - 0.707) |
| Age |  | 1.001 | 0.999 |  | 1.003 | 1.002 |
|  |  | (0.997 - 1.004) | (0.994 - 1.005) |  | (0.999 - 1.006) | (0.996 - 1.008) |
| Marital Status (Base: Currently Married) | | | |  |  |  |
| Never Married |  | 1.421*** | 1.194** |  | 1.378*** | 1.187* |
|  |  | (1.277 - 1.582) | (1.003 - 1.421) |  | (1.221 - 1.555) | (0.986 - 1.429) |
| Widow / Widower |  | 0.900 | 0.631** |  | 0.930 | 0.719 |
|  |  | (0.725 - 1.118) | (0.410 - 0.971) |  | (0.731 - 1.182) | (0.451 - 1.148) |
| Divorced |  | 0.851 | 0.595 |  | 1.237 | 1.116 |
|  |  | (0.444 - 1.631) | (0.238 - 1.489) |  | (0.553 - 2.766) | (0.407 - 3.062) |
| Separated |  | 1.035 | 1.024 |  | 1.099 | 1.361 |
|  |  | (0.438 - 2.441) | (0.274 - 3.823) |  | (0.431 - 2.803) | (0.340 - 5.441) |
| Married but lives with parents |  | 0.988 | 1.046 |  | 1.191 | 1.212 |
|  |  | (0.558 - 1.749) | (0.482 - 2.267) |  | (0.595 - 2.384) | (0.488 - 3.006) |
| Education (Base: No Education) | | |  |  |  |  |
| Nursery |  | 0.575*** | 0.748 |  | 0.797 | 0.923 |
|  |  | (0.392 - 0.845) | (0.390 - 1.437) |  | (0.503 - 1.260) | (0.441 - 1.933) |
| Kindergarten |  | 1.269*** | 1.254 |  | 1.491*** | 1.656*** |
|  |  | (1.080 - 1.492) | (0.900 - 1.746) |  | (1.239 - 1.794) | (1.151 - 2.383) |
| Primary |  | 1.060 | 0.922 |  | 1.156** | 1.011 |
|  |  | (0.945 - 1.188) | (0.762 - 1.114) |  | (1.018 - 1.314) | (0.827 - 1.236) |
| Middle |  | 1.126* | 1.135 |  | 1.462*** | 1.442*** |
|  |  | (0.988 - 1.284) | (0.924 - 1.395) |  | (1.262 - 1.694) | (1.158 - 1.794) |
| Matric |  | 1.154** | 1.024 |  | 1.472*** | 1.321*** |
|  |  | (1.020 - 1.305) | (0.854 - 1.229) |  | (1.283 - 1.689) | (1.090 - 1.601) |
| Intermediate |  | 1.245*** | 1.152 |  | 1.605*** | 1.647*** |
|  |  | (1.067 - 1.453) | (0.912 - 1.455) |  | (1.350 - 1.908) | (1.286 - 2.108) |
| Engineering |  | 1.407 | 1.550 |  | 2.685** | 3.330** |
|  |  | (0.739 - 2.679) | (0.584 - 4.119) |  | (1.256 - 5.741) | (1.124 - 9.869) |
| Medicine |  | 1.580 | 1.932 |  | 2.637* | 2.609 |
|  |  | (0.617 - 4.045) | (0.564 - 6.613) |  | (0.957 - 7.266) | (0.745 - 9.133) |
| Computer Science |  | 2.847 | 1.941 |  | 6.727* | 5.329 |
|  |  | (0.571 - 14.204) | (0.300 - 12.544) |  | (0.973 - 46.520) | (0.576 - 49.323) |
| Agriculture |  | - | - |  | - | - |
|  |  |  |  |  |  |  |
| Other Subjects |  | 1.106 | 1.144 |  | 1.562*** | 1.615*** |
|  |  | (0.905 - 1.352) | (0.862 - 1.519) |  | (1.251 - 1.950) | (1.198 - 2.175) |
| MSc |  | 1.547*** | 1.400** |  | 2.319*** | 1.917*** |
|  |  | (1.180 - 2.027) | (1.001 - 1.957) |  | (1.716 - 3.133) | (1.347 - 2.727) |
| M.Phils. |  | 2.279 | 2.182 |  | 3.577* | 2.349 |
|  |  | (0.659 - 7.885) | (0.470 - 10.141) |  | (0.929 - 13.780) | (0.503 - 10.970) |
| PhD |  | 1.021 | 1.518 |  | 0.837 | 1.273 |
|  |  | (0.361 - 2.884) | (0.377 - 6.119) |  | (0.277 - 2.526) | (0.284 - 5.716) |
| Rural (Base: Urban) |  | 0.792*** | 0.873** |  | 0.608*** | 0.616*** |
|  |  | (0.733 - 0.856) | (0.767 - 0.994) |  | (0.558 - 0.664) | (0.537 - 0.706) |
| Province (Base: Punjab) | |  |  |  |  |  |
| KPK |  | 0.168*** | 0.187*** |  | 0.177*** | 0.200*** |
|  |  | (0.149 - 0.188) | (0.155 - 0.226) |  | (0.155 - 0.202) | (0.163 - 0.245) |
| Sindh |  | 0.422*** | 0.543*** |  | 0.361*** | 0.440*** |
|  |  | (0.379 - 0.470) | (0.460 - 0.641) |  | (0.320 - 0.407) | (0.369 - 0.524) |
| Baluchistan |  | 0.295*** | 0.114*** |  | 0.119*** | 0.062*** |
|  |  | (0.261 - 0.334) | (0.094 - 0.139) |  | (0.104 - 0.136) | (0.051 - 0.076) |
| Gilgit-Baltistan |  | 0.194*** | 0.174*** |  | 0.191*** | 0.197*** |
|  |  | (0.164 - 0.230) | (0.133 - 0.227) |  | (0.157 - 0.232) | (0.147 - 0.264) |
| AJ&K |  | 0.396*** | 0.715* |  | 0.324*** | 0.590** |
|  |  | (0.327 - 0.480) | (0.490 - 1.045) |  | (0.263 - 0.399) | (0.394 - 0.883) |
| ln (Monthly Income) |  |  | 1.009 |  |  | 1.038*** |
|  |  |  | (0.987 - 1.031) |  |  | (1.015 - 1.062) |
| Constant | 4.153*** | 14.555*** | 12.463*** | 4.490*** | 14.887*** | 10.547*** |
|  | (4.006 - 4.307) | (12.184 - 17.386) | (8.781 - 17.689) | (4.316 - 4.671) | (12.182 - 18.193) | (7.284 - 15.271) |
| Observations | 22,616 | 22,611 | 7,827 | 22,616 | 22,611 | 7,827 |
| Adjustment Variables | No | Yes | Yes | No | Yes | Yes |
| Extended Adjustment Variable | No | No | Yes | No | No | Yes |
| Pseudo R-Squared | 0.140 | 0.212 | 0.244 | 0.276 | 0.349 | 0.320 |

95% Confidence intervals in parentheses. *** p<0.01, ** p < 0.05, * p <0.10. Varimax rotation is performed during PCA for both indices.
